# Molecular Theranostics in Radioiodine-Refractory Differentiated Thyroid Cancer

**DOI:** 10.3390/cancers15174290

**Published:** 2023-08-27

**Authors:** Petra Petranović Ovčariček, Alfredo Campenni, Bart de Keizer, Desiree Deandreis, Michael C. Kreissl, Alexis Vrachimis, Murat Tuncel, Luca Giovanella

**Affiliations:** 1 Department of Oncology and Nuclear Medicine, University Hospital Center Sestre Milosrdnice, 10000 Zagreb, Croatia; 2 School of Medicine, University of Zagreb, 10000 Zagreb, Croatia; 3 Department of Biomedical and Dental Sciences and Morpho-Functional Imaging, University of Messina, 98100 Messina, Italy; alfredo.campenni@unime.it; 4 Department of Nuclear Medicine and Radiology, University Medical Center Utrecht, 3584 CX Utrecht, The Netherlands; B.dekeizer@umcutrecht.nl; 5 Institute Gustave Roussy, 94800 Villejuif, France; desiree.deandreis@gustaveroussy.fr; 6 Division of Nuclear Medicine, Department of Radiology and Nuclear Medicine, University Hospital Magdeburg, Otto-von-Guericke University, 39120 Magdeburg, Germany; michael.kreissl@med.ovgu.de; 7 Department of Nuclear Medicine, German Oncology Center, University Hospital of the European University, Limassol 4108, Cyprus; alexis.vrachimis@goc.com.cy; 8 Department of Nuclear Medicine, Hacettepe University, Ankara 06230, Turkey; murat.tuncel@hacettepe.edu.tr; 9 Clinic for Nuclear Medicine, Imaging Institute of Southern Switzerland, Ente Ospedaliero Cantonale, 6500 Bellinzona, Switzerland; luca.giovanella@eoc.ch; 10 Clinic for Nuclear Medicine, University Hospital of Zürich, 8004 Zürich, Switzerland

**Keywords:** radioiodine-refractory DTC, molecular imaging, FDG, PSMA, FAPI, somatostatin analogues, PRRT, radioligand therapy, theranostics

## Abstract

**Simple Summary:**

Radioiodine therapy is the main treatment option for metastatic differentiated thyroid cancer (DTC). However, only half of these patients achieve (partial or complete) remission or have stable disease during long-term follow-up. In the remaining ones, disease progresses mainly as they become radioiodine-refractory. The diagnostics of radioiodine-refractory disease are extensively debated. The introduction of novel tracers besides radioiodine isotopes (^131^I, ^123^I, and ^124^I) and 2-[^18^F]fluoro-2-deoxy-D-glucose ([^18^F]FDG) opens new options for the diagnostics and therapy of this subgroup of DTC patients. Prostate-specific membrane antigen (PSMA) ligands, fibroblast activation protein inhibitors (FAPI), and somatostatin receptor-targeted radiopharmaceuticals appear to be new potential theranostics tracers. In this review, we will elaborate on the role of these radiopharmaceuticals in the management of radioiodine-refractory disease.

**Abstract:**

Differentiated thyroid cancer (DTC) is the most common subtype of thyroid cancer and has an excellent overall prognosis. However, metastatic DTC in certain cases may have a poor prognosis as it becomes radioiodine-refractory. Molecular imaging is essential for disease evaluation and further management. The most commonly used tracers are [^18^F]FDG and isotopes of radioiodine. Several other radiopharmaceuticals may be used as well, with different diagnostic performances. This review article aims to summarize radiopharmaceuticals used in patients with radioiodine-refractory DTC (RAI-R DTC), focusing on their different molecular pathways. Additionally, it will demonstrate possible applications of the theranostics approach to this subgroup of metastatic DTC.

## 1. Introduction

The 5-year disease-specific survival in DTC patients is excellent in those with localized and regional disease (above 98%). However, it is significantly lower in patients with distant metastases (approximately 50%) [1]. Radioiodine therapy is the cornerstone of metastatic DTC, which accounts for 10% of patients. Half of the metastatic patients achieve complete or partial remission or have stable disease over a long period following radioiodine therapy. Unfortunately, in the remaining patients, the disease progresses despite the therapy [2]. Post-therapeutic radioiodine whole-body imaging is crucial for staging and evaluating radioiodine avidity in recurrent or metastatic disease [3]. Approximately 70% of patients with metastatic disease demonstrate radioiodine uptake, whereas the remaining develop non-radioiodine-avid metastases or have progressive disease despite radioiodine treatment [3].

There is increasing evidence that treatment response in advanced and metastatic disease is related to tumor-absorbed doses, leading to the need for personalized therapy. Beyond iodine-131 (^131^I) uptake, several other factors, such as the molecular pathogenesis and mechanisms of DTC, specific patients’ characteristics, and disease presentation, should be taken into account and considered on an individual basis [4].

Imaging has a crucial role in the diagnostics, therapeutic approaches, and monitoring of radioiodine-refractory (RAI-R) DTC patients. Computed tomography (CT) and magnetic resonance imaging (MRI) are anatomical imaging modalities that may be used for the detection of residual or recurrent disease in patients with high serum thyroglobulin (Tg) [1]. Nuclear medicine functional imaging, i.e., single photon emission computed tomography (SPECT) and positron emission tomography (PET), in combination with CT or MRI, besides anatomical, provides the evaluation and quantification of DTC lesions at the molecular level.

This review article aims to summarize currently used radiopharmaceuticals in RAI-R DTC, their molecular uptake mechanisms, and their possible use in therapy, i.e., theranostics.

## 2. Radioiodine-Refractory Differentiated Thyroid Cancer: Definition and Criteria

Among DTC patients, about 30% have or will develop metastatic disease at loco-regional lymph nodes (5-year survival rate > 90%) or, more rarely, in distant organs (mainly lungs and bones) with a significantly worse prognosis (5-year survival approx. 55%). Additionally, an increase in the overall mortality rate of DTC patients has been observed lately, probably due to an increased incidence of metastatic DTC patients [5]. Luckily, many patients with advanced DTC still exhibit ^131^I avidity, and therefore approximately 40% will achieve remission after ^131^I treatment [6,7,8,9]. Consequently, repeated courses of ^131^I therapy, in addition to TSH suppression, are the standard of care to manage metastasized DTC when the disease remains iodine-avid [10,11].

Unfortunately, ^131^I therapy becomes ineffective in a fraction of patients and should be stopped when a patient no longer responds to treatment. However, considering that RAI-R DTC patients still have limited therapeutic options, premature stopping of ^131^I therapy (i.e., when still able to obtain disease stabilization and symptom relief) should be avoided.

The correct identification of RAI-R disease remains controversial (Table 1). Currently, different criteria to define an RAI-R disease are reported in the literature. In 2014, an international expert panel proposed stopping ^131^I therapy when “at least one lesion becomes ^131^I negative and continues to grow” [2]. Sacks and colleagues supported stopping ^131^I therapy when diagnostic scintigraphy (i.e., low ^131^I or ^123^I activity administered) is negative in the presence of structural disease, in the case of a positive [^18^F]FDG PET scan or cumulative administered activities of >18.5–22 GBq ^131^I [12].

Finally, the American Thyroid Association 2015 Guidelines propose criteria summarized in Recommendation 91. “Radioiodine-refractory, structurally evident DTC is classified in patients with appropriate TSH stimulation and iodine preparation in four basic ways: (i) the malignant/metastatic tissue does not ever concentrate RAI (no uptake outside the thyroid bed at the first therapeutic WBS); (ii) the tumor tissue loses the ability to concentrate RAI after previous evidence of RAI-avid disease (in the absence of stable iodine contamination); (iii) RAI is concentrated in some lesions but not in others; and (iv) metastatic disease progresses despite significant concentration of RAI” [3].

Furthermore, the BRAFV600E gene mutation seems to exhibit more aggressive tumor behavior and is more often associated with RAI-R disease; it also carries an increased risk of recurrence and a higher disease-specific mortality [13].

However, as discussed by Giovanella and van Nostrand, any classification is only conditionally appropriate for managing individualized patient care [11]. Notably, the absence of visualization of malignant tissues on a diagnostic and/or post-therapy whole-body scintigraphy greatly increases the likelihood that DTC metastases are RAI-R. However, a careful standardization of imaging in terms of preparation, prescribed activity, and imaging technique is pivotal to avoiding false-negative results and, consequently, an inappropriate discontinuation of (still active) ^131^I therapy [14,15].

After assessment of multiple factors and aiming to personalize the patient’s care, at the time that a patient is declared RAI-R, ^131^I therapy is discontinued and alternative therapies are considered. A careful staging of RAI-R disease is integral to deciding on further therapeutic strategies, monitoring treatment efficacy, and detecting progressive disease. In addition to conventional cross-sectional imaging (i.e., CT, MRI), various positron-emitting radiopharmaceuticals enable the use of PET in combination with CT (PET/CT) or MR (PET/MR) to characterize different biological and molecular characteristics of RAI-R disease. This is highly relevant to personalizing the management of RAI-R disease, and, in addition, some radiopharmaceuticals open the door to theranostics applications by using companion beta- or alpha-emitting radiopharmaceuticals. 

## 3. Radiopharmaceuticals Used for Radioiodine-Refractory Differentiated Thyroid Cancer Imaging and Therapy

### 3.1. [^18^F]FDG

[^18^F]FDG is, besides radioiodine, the most commonly employed molecular imaging tracer in RAI-R DTC. It is considered especially appropriate in DTC patients with elevated serum Tg and negative radioiodine whole-body imaging [16], and its sensitivity depends on tumor differentiation, which has a superior detection rate in DTC patients with aggressive histopathology/biological behavior.

**Figure 1:**
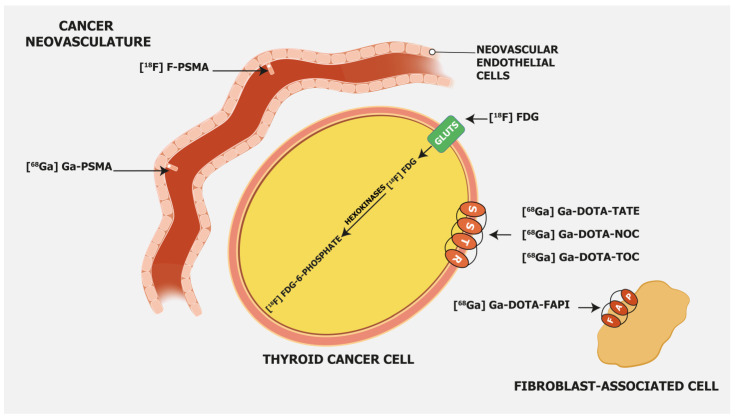
Mechanism of uptake of different radiopharmaceuticals in radioiodine-refractory differentiated thyroid cancer. Legend: FAP—fibroblast activation protein; GLUT—glucose transporter; SSTR—somatostatin receptors; [^18^F]FDG—2-[^18^F]fluoro-2-deoxy-D-glucose; [^68^Ga]Ga-PSMA—[^68^Ga]gallium-prostate-specific membrane antigen; [^18^F]F-PSMA—[^18^F]fluoro-prostate-specific membrane antigen; [^68^Ga]Ga-DOTA-TATE—[^68^Ga]Gallium-DOTA-Tyr^3^-octreotate; [^68^Ga]Ga-DOTA-NOC—[^68^Ga]Gallium-DOTA-NaI^3^-octreotide; [^68^Ga]Ga-DOTA-TOC—[^68^Ga]Gallium-DOTA-Tyr^3^-octreotide; [^68^Ga]Ga-DOTA-FAPI- [^68^Ga]Gallium-DOTA-fibroblast activation protein inhibitor.

[^18^F]FDG, as a glucose analogue, is transported into the cells across the glucose transporter (GLUT) protein and phosphorylated by hexokinase into [^18^F]FDG-6-phosphate, which is not metabolized but trapped within the cell (Figure 1).

Cancer cells have a higher concentration of membranous GLUT proteins, such as GLUT1 and GLUT3, and more enzymes involved in the glycolytic pathway, which is even more pronounced in undifferentiated cancer cells. Therefore [^18^F]FDG is more intensely accumulated in cancer cells as compared to normal cells [17]. In undifferentiated DTC cells, iodine accumulation is decreased or lost, but GLUT proteins are upregulated, and consequently, [^18^F]FDG uptake is higher [18]. Feine et al. demonstrated the “flip-flop” phenomenon, i.e., the reverse uptake of ^131^I and [^18^F]FDG in metastatic DTC patients. Patterns marked with ^131^I-negative and [^18^F]FDG-positive uptake represent the most common type in patients with increased Tg and negative ^131^I scintigraphy [19,20]. A prospective study that included 122 metastatic DTC patients demonstrated an association between [^18^F]FDG uptake and loss of iodine avidity. The maximum standardized uptake value (SUVmax) cut-off of 4.0 provided a sensitivity and a specificity of 75.3% and 56.7%, respectively, for identifying ^131^I-negative metastatic disease [21].

In a clinical setting, [^18^F]FDG PET/CT is useful as a prognostic factor for RAI treatment response in metastatic DTC patients. High [^18^F]FDG uptake is an independent negative prognostic factor for overall survival [22]. A retrospective study that enrolled 80 patients with metastatic DTC showed that [^18^F]FDG uptake is the only significant prognostic factor for disease-specific survival. A higher SUVmax and a larger number of [^18^F]FDG-avid metastases were also associated with a poor prognosis. Additionally, patients with [^18^F]FDG and RAI avid lesions had a similar prognosis as those with [^18^F]FDG-only avid metastases [23]. A recent study that involved 62 patients with RAI-refractory metastatic DTC showed that [^18^F]FDG is a predictive biomarker for progression-free survival (PFS) and overall survival (OS). Higher than median values of metabolic tumor volume (MTV) and total lesion glycolysis (TLG) correlated with worse PFS and OS. Additionally, a higher risk of death was demonstrated in patients with lesions having higher values of log-MTV and log-TLG [24]. Albano et al., in a study that included 122 patients with [^18^F]FDG-positive/^131^I-negative lesions and Tg > 10 ng/mL, found that the total MTV and total TLG were the only independent prognostic factors for OS, and the best cut-offs for MTV and TLG were 6.6 cm^3^ and 119.4, respectively [25]. [^18^F]FDG PET could also predict patients with certain clinicopathological features and aggressive mutations that are reported to be related to radioiodine refractoriness [26,27].

A meta-analysis by Santhanam and colleagues showed that DTC patients with a BRAF^V600E^ mutation have a higher likelihood of having [^18^F]FDG-avid disease, with a pooled odds ratio (OR) of 2.12. The pooled mean SUV value was significantly higher in BRAF^V600E^-positive patients, with a pooled mean difference of 5.1, compared to BRAF^V600E^-negative DTC patients [28]. 

[^18^F]FDG PET/CT can also be used for follow-up and to predict the success of redifferentiation therapies with BRAF, MEK, or RET inhibitors [29,30]. Weber et al. used MAPK inhibitors in a prospective study to enhance radioiodine uptake in RAI-R DTC (ERRITI trial) [30]. They demonstrated that the peak SUV < 10.0 on [^18^F]FDG PET/CT is associated with successful redifferentiation, presumably due to the known positive correlation between [^18^F]FDG uptake and dedifferentiation of the tumor.

Regarding the optimal preparation protocol for [^18^F]FDG imaging on levothyroxine vs. after stimulation with recombinant human thyrotropin (rhTSH), there is still an academic discussion ongoing. Leboulleux and colleagues performed a prospective study with 63 DTC patients (52 with papillary thyroid cancer and 11 with follicular thyroid cancer). All patients underwent both basal and rhTSH-stimulated (24 and 48 h before tracer administration) [^18^F]FDG PET/CT. The colleagues found that the per-patient sensitivity was not different between basal and rhTSH-stimulated imaging studies [31]. On the other hand, the use of rhTSH significantly increased the per-lesion sensitivity (i.e., the number of detected lesions). However, this resulted in a change of treatment plan in only 6% of the cases.

### 3.2. PSMA-Targeting Radiopharmaceuticals

**Figure 2:**
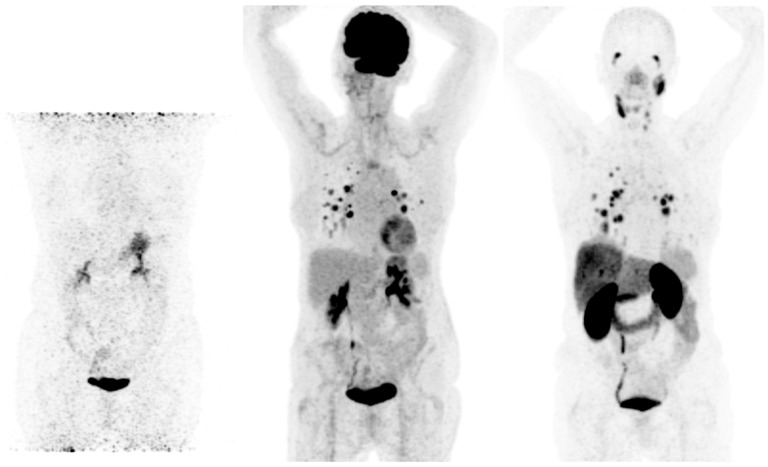
Radioiodine-refractory metastatic differentiated thyroid cancer with positive lesions on [^18^F]FDG PET and [^68^Ga]Ga-PSMA PET Legend: example of a 65-year-old female patient with lung metastases and lymph node neck metastases maximum intensity projection (MIP) of ^124^I (**left**), [^18^F]FDG PET (**middle**), and [^68^Ga]Ga-PSMAPET (**right**). No uptake is seen in the metastases on ^124^I PET, and high uptake is seen in lung metastases on [^18^F]FDG and [^68^Ga]Ga-PSMA PET. On [^68^Ga]Ga-PSMA PET, lymph node metastases on the left side of the neck are also visible. The patient was treated with 2 cycles of 6 GBq [^177^Lu]Lu-PSMA-617, unfortunately without an objective response.

Prostate-specific membrane antigen (PSMA) is a transmembrane glycoprotein type II encoded by the Folate Hydrolase 1 gene [32] expressed in prostate cancer but also on the membrane of neovascular endothelial cells of various solid tumors, such as thyroid, head, bladder, lung, breast, gynecologic, gastric, and colorectal cancers [33] (Figure 1). Immunohistochemical studies have demonstrated that high PSMA expression in the neovasculature of thyroid cancer positively correlates with a more clinically aggressive course of the disease. Moreover, it was shown that DTC patients with lesions of moderate and strong PSMA expression have a higher risk of developing radioiodine-refractory disease or a higher risk of disease-specific mortality [34,35] (Figure 2).

This finding opens the door for the use of PSMA-targeting tracers for diagnostic but also therapeutic purposes. PSMA-targeting tracers are usually labeled with ^68^Ga and ^18^F. From a technical point of view, PET performs better with ^18^F as compared to ^68^Ga due to the shorter positron range, lower maximum β^+^energy, and higher positron yield, which leads to better spatial resolution [36]. ^68^Ga has an 88% abundance of positron emission and a higher maximum energy than ^18^F, which gives more noise to images and leads to a lower resolution [37]. Other major advantages of ^18^F over ^68^Ga-labeled PSMA tracers are the longer physical half-life of ^18^F and its higher availability. In the end, it leads to fewer technical challenges. However, due to defluorination, ^18^F-labeled PSMA compounds have higher bone uptake [38,39]. A recent multicenter study that enrolled 348 prostate cancer patients demonstrated unspecific bone uptake of [^18^F]F-PSMA-1007 in 217 (51.4%) patients, of which in 80 (44.7%) patients it was crucial for further therapeutic approaches and was considered clinically important [40]. Similar results were found in a recent retrospective study that included 214 patients. Ninety-four (43.9%) patients had at least one non-specific bone lesion. An SUVmax cut-off of 7.2 was set to distinguish between benign and metastatic lesions [41].

Besides PET tracers, there are also more affordable ^99m^Tc-labeled PSMA SPECT tracers. However, they have lower sensitivity due to the lower performance of SPECT compared to PET technology. Still, a very recent Australian study demonstrated that [^99m^Tc]Tc-PSMA SPECT/CT with an improved reconstruction algorithm has a diagnostic performance similar to [^68^Ga]Ga-PSMA PET/CT in a daily clinical setting [42].

Recent studies examined the performance of PSMA-targeting radiopharmaceuticals in DTC patients. Rizzo et al. performed a systematic review of six studies, including 49 patients, to evaluate the diagnostic performance of PSMA-targeted radiopharmaceuticals in RAI-R DTC [43]. Reported detection rates varied significantly from 25% to 100% and were overall lower as compared to [^18^F]FDG PET/CT in comparative studies. On a per-patient-based analysis, detection rates in 4 studies were 25–83%, whereas on a lesion-based analysis, they were around 65%. Only two studies demonstrated a 100% detection rate on the patient-based analysis. Additionally, heterogeneity between SUVmax values was also observed in the per-lesion analysis (1.0–39.7).

Diagnostic PSMA-targeted tracers are crucial to evaluating potential candidates for radioligand therapy. Theoretically, only those with significant uptake in tumor lesions are suitable to undergo PSMA-targeted radioligand therapy. Two studies reported the use of [^177^Lu]Lu-PSMA-targeted therapies in three patients. Two of the three included patients had a temporary partial response to therapy, while in the third case, the disease progressed one month following the treatment [44,45]. Regarding the side effects, temporary nausea was noted after the second course of therapy in one patient [45].

In comparison to [^18^F]FDG PET/CT, PSMA-targeted tracers have a lower detection rate, and their use generally does not affect further patient management, at least according to the currently available data in a limited number of patients [43]. Therefore, in its current setting, PSMA-targeted diagnostics cannot be suggested as an alternative imaging method for restaging patients with RAI-R DTC. 

Wächter and colleagues investigated the correlation between the expression of PSMA on histological samples and PSMA-positive lesions on PET/CT scans. Surprisingly, only three patients had concordant results. Ciappuccini et al. evaluated PSMA expression in 44 DTC patients with persistent and/or recurrent neck disease using immunohistochemistry [33]. Expression of PSMA was quantified using the immunoreactive score (IRS). Approximately 68% of the patients demonstrated at least one PSMA-positive lesion showing IRS ≥ 2, i.e., 66% and 75% in RAI-negative patients and RAI-positive patients, respectively. In RAI-negative patients, higher expression of PSMA was found in [^18^F]FDG-positive (79%) patients as compared to [^18^F]FDG-negative patients (25%), and the mean value of IRS was higher in [^18^F]FDG-positive (4.0) patients as compared to [^18^F]FDG-negative patients (1.0). In addition, patients ≥ 55 years old, with a primary tumor > 40 mm, or with an aggressive subtype had a higher mean IRS. Strong expression of PSMA, i.e., IRS ≥ 9, was correlated with shorter progression-free survival. Further studies on a molecular level are needed to evaluate these contradictory results.

Although the application of [^177^Lu]Lu-PSMA-targeting radioligand therapy in treating DTC patients has been limited, parallels can be drawn with the experiences of prostate cancer patients in terms of toxicity [46]. If [^177^Lu]Lu-PSMA treatment is administered to individuals with thyroid cancer, the likelihood of encountering xerostomia is probably further elevated due to potential residual damage to the thyroid glands from prior ^131^I therapies.

The literature data in the setting of radioiodine-refractory DTC are limited. Currently, the limited use of PSMA-targeting radiopharmaceuticals does not significantly affect further patient management. Prospective multicentric studies are needed to evaluate its potential role in RAI-R DTC patients.

### 3.3. Somatostatin Receptor-Targeting Radiopharmaceuticals

**Figure 3:**
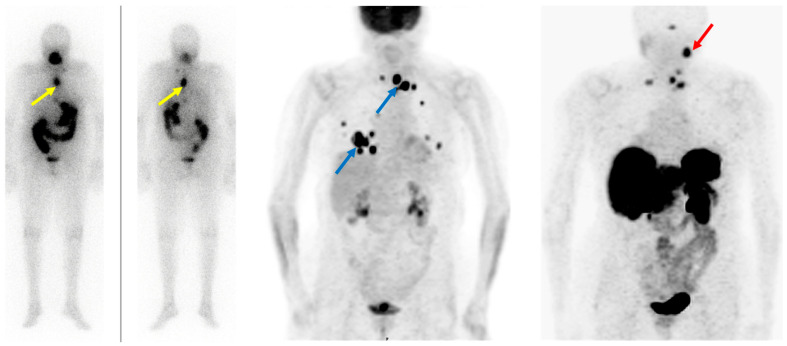
Radioiodine-refractory metastatic differentiated thyroid cancer with positive lesions on [^18^F]FDG PET and [^68^Ga]Ga-DOTANOC PET. Legend: Eighty-four-year-old patient with advanced follicular thyroid cancer and (new) pulmonary and lymph node metastases, after 17 GBq ^131^I, Tg: 8660 ng/mL. (**Left**): diagnostic ^131^I imaging showing radioiodine-avid lymph node metastases (yellow arrows) in the chest. (**Middle**): [^18^F]FDG PET depicts discordance with RAI-imaging lymph node metastases in the upper mediastinum and lung metastases (blue arrows). (**Right**): [^68^Ga]Ga-DOTANOC PET shows SSTR-expression in some but not all lymph node metastases in the upper mediastinum; additionally, visualization of a lesion in the left neck not seen with [^18^F]FDG and ^131^I (red arrow). However, no SSTR expression in the lung metastases was observed.

Somatostatin receptor-targeting radiopharmaceuticals are used for imaging tumors with high expression of somatostatin receptors (SSTR) (Figure 1). Most commonly, somatostatin analogs are labeled with ^68^Ga, such as [^68^Ga]Ga-DOTA-TATE, [^68^Ga]Ga-DOTA-NOC, and [^68^Ga]Ga-DOTA-TOC. They have different binding affinities for different SSTR subtypes. [^68^Ga]Ga-DOTA-TATE has a quite selective and very high affinity for SSTR 2, [^68^Ga]Ga-DOTA-NOC binds SSTR 2 and 3, while [^68^Ga]Ga-DOTA-TOC has an affinity for SSTR 2 and SSTR 5 [47]. DTC cells may exhibit high expression of SSTR 2, 3, and 5 [48,49] (Figure 3).

Therefore, radiolabeled somatostatin analogs may detect DTC recurrence or metastases, which are especially noted in patients with RAI-refractory disease. Ocak and colleagues enrolled 13 patients with RAI-refractory DTC (nine with papillary thyroid cancer, one with follicular thyroid carcinoma, and three with Hurthle cell carcinoma) to evaluate and compare the performance of [^68^Ga]Ga-DOTA-TATE and [^68^Ga]Ga-DOTA-NOC in the detection of RAI-R DTC lesions [50]. Somatostatin-positive lesions were found in eight (62%) patients. Forty-five lesions were detected with [^68^Ga]Ga-DOTA-TATE and 42 with [^68^Ga]Ga-DOTA-NOC. Lesion uptake was significantly higher on [^68^Ga]Ga-DOTA-TATE (SUVmax 12.9 ± 9.1) compared to [^68^Ga]Ga-DOTA-NOC (SUVmax 6.3 ± 4.1), suggesting its potential advantage in RAI-R DTC imaging.

Positive RAI-R DTC lesions on somatostatin receptor imaging open the possibility of treating these patients with peptide receptor radionuclide therapy (PRRT) based on the theranostic approach. The theranostic approach with radiolabeled somatostatin analogs is important in the management of metastatic SSTR-positive tumors, nowadays mostly neuroendocrine tumors. ^177^Lu-labeled or ^90^Y-labeled somatostatin analogs are the most common therapeutic radiopharmaceuticals.

Versari and colleagues used [^68^Ga]Ga-DOTA-TOC PET/CT to select patients for PRRT and to evaluate treatment response and its toxicity in RAI-R DTC patients [51]. They enrolled 41 patients, out of which 24 were [^68^Ga]Ga-DOTA-TOC positive. Thirteen patients demonstrated high expression of SSTR, and 11 underwent PRRT with [^90^Y]Y-DOTATOC according to additional inclusion criteria. Partial response and stable disease were induced in approximately 64% of patients (7/11) during the 3.5–11.5-month period. The main adverse events were transient hematologic toxicity, nausea, and asthenia, while one patient experienced permanent renal toxicity.

Maghsoomi et al., in a recent meta-analysis, evaluated the efficacy and safety of PRRT in patients with advanced RAI-R DTC and metastatic medullary thyroid carcinoma (MTC). Forty-one out of 2284 related papers were included in the analysis. One hundred fifty-seven RAI-R DTC patients had undergone PRRT. Biochemical response was determined in 25.3% of patients, while objective response, both partial and complete, was noted in 10.5% of patients [52]. The most common side effects were nausea, asthenia, and increased liver enzymes, while major side effects, e.g., nephrotoxicity and hematologic adverse effects, were rare and mostly transient.

A recent meta-analysis by Lee and colleagues also evaluated the therapeutic effect of PRRT in metastatic RAI-R, DTC, and MTC [53]. They involved 67 DTC patients. The pooled objective response rate was 8.5–15.6%, the disease control rate was 54–60.0%, and major adverse effects were observed in 2.79–2.82% of cases. ^177^Lu-based somatostatin analogs (ORR, 11.48–24.52%; DCR, 61.47–67.26%) demonstrated better therapeutic efficiency than ^90^Y-based analogs (ORR, 6.98–13.82%; DCR, 50.86–57.29%).

Budiawan et al. studied the toxicity of ^90^Y and ^177^Lu-labeled somatostatin analogs in 16 RAI-R DTC patients receiving 45 PRRT courses [54]. Hematological and kidney functions were evaluated following the treatment. Only mild and reversible hematotoxicity (grade 1) and nephrotoxicity (grade 1) were detected. An elevation in liver enzymes of grades 1–3 (only one case with a grade 3 elevation, but a reversible one) was observed in six patients.

Despite the heterogeneous response, PRRT may be an alternative treatment option for advanced and metastatic RAI-R DTC with sufficient expression of SSTRs owing to its efficacy and promising safety profile, especially in those patients experiencing progression under the standard treatment options, i.e., tyrosine kinase inhibitors.

### 3.4. Fibroblast Activation Protein—Targeting Radiopharmaceuticals

Fibroblast activation protein (FAP) expression is very low in normal human fibroblasts. However, cancer-related fibroblasts are characterized by high expression of FAP (Figure 1) since they carry both exopeptidase and endopeptidase activity [17]. A large fraction of the total mass in various tumors is made of tumor fibroblasts and extracellular fibrosis, while on many occasions less than 10% of tumor cells are involved [55]. Thus, radiolabeled fibroblast activation protein inhibitors (FAPIs) are suitable for tumor imaging.

Chen et al. recently conducted a study to evaluate the performance of [^68^Ga]Ga-DOTA-FAPI-04 PET/CT in the detection of RAI-R DTC lesions [56]. They enrolled 24 RAI-R DTC patients and demonstrated that 21 (87.5%) patients have FAPI-positive lesions, with a mean SUVmax of 4.25 and a growth rate of 6.51%. SUVmax was positively correlated with the lesions’ growth rates.

In certain cases, [^68^Ga]Ga-DOTA-labeled FAPI radiopharmaceuticals showed better detection of metastatic RAI-R DTC lesions compared to [^18^F]FDG, also due to a better target-to-background ratio [57]. Detectable strong expression of FAP opens an opportunity for new therapeutic options in RAI-R DTC patients.

**Figure 4:**
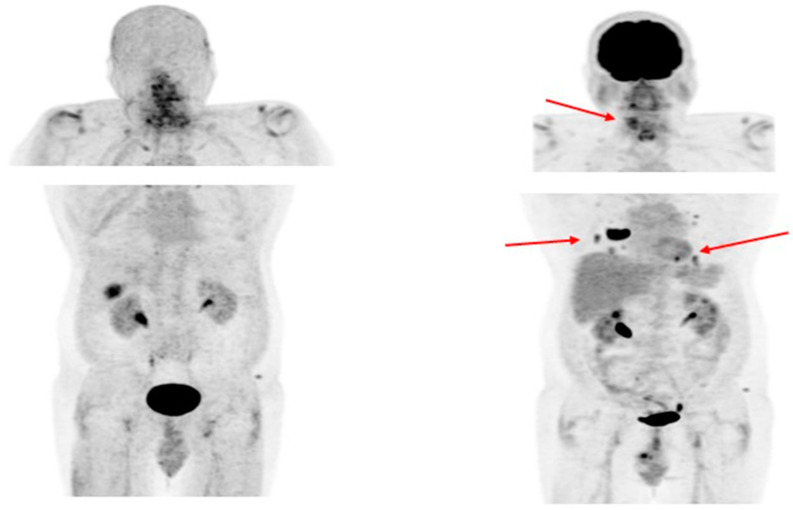
Radioiodine-refractory metastatic differentiated thyroid cancer with positive lesions on [^18^F]FDG PET and negative [^18^F]FAPI-74 PET. Legend: Sixty-four-year-old patient with RAI-negative (not shown) papillary thyroid cancer after 15 GBq ^131^I. (**Left**): [^18^F]FAPI-74 PET shows a physiological tracer distribution. (**Right**): [^18^F]FDG PET depicts in discordance with [^18^F]FAPI-74 local relapse and multiple pulmonary metastases (red arrows).

Like PSMA labeling, several attempts have been made for ^18^F labeling of FAPI ligands to overcome ^68^Ga limitations as stated above (e.g., longer positron range, higher maximum ^+^energy, lower positron yield, higher costs, etc.), with [^18^F]FAPI-74 being the most promising candidate. Studies are currently ongoing, e.g., for the diagnostic comparison of [^18^F]FDG with [^18^F]FAPI-74 in patients with TENIS syndrome (EudraCT Number: 2022-001997-70; Figure 4). 

Ballal et al. performed a study to evaluate the efficiency and safety of [^177^Lu]Lu-DOTAGA.(SA.FAPi)_2_ in metastatic RAI-R DTC patients who progressed on TKI therapy (sorafenib/lenvatinib) [58]. They prospectively enrolled 15 metastatic patients who had moderate-to-strong uptake of [^68^Ga]Ga-DOTA.SA.FAPi. Serum Tg levels significantly decreased following the treatment with [^177^Lu]Lu-DOTAGA.(SA.FAPi)_2_. The baseline median Tg level was 10,549 ng/mL, while posttherapy it was 5649 ng/mL. Although there was no molecular complete response, partial responses were achieved in four patients and stable disease in three RAI-R DTC patients. The maximal visual analog score before the therapy was 9, while on post-therapeutic imaging it was 6. Likewise, Eastern Cooperative Oncology Group performance status was initially 3, and after the therapy it was 2, thus showing significant improvement after the [^177^Lu]Lu-DOTAGA.(SA.FAPi)_2_ treatment. In the safety profile evaluation, there was no grade III or IV hematological, renal, or liver toxicity detected. The results demonstrate that FAPI-based theranostics could provide an additional treatment option for patients with metastatic RAIR-DTC who progress on standard therapeutic strategies.

## 4. Tumor Heterogeneity

Tumorigenesis usually starts with a single mutated cell, evolving with sequential clonal mutations leading to the occurrence of genetically distinct tumor subpopulations. Cancers generally become more heterogeneous with a longer duration of the disease, and intratumoral heterogeneity is a result of the accumulation of mutations during the progression of the disease [59]. Different cell clones with differing molecular signatures are observed in many cancer patients during follow-up. These undesirable events result in a heterogeneous distribution of tumor clones in different disease sites and even within the same lesion. Various tumor subclones may further influence the course of the disease, as some may be more aggressive than others. All subclones usually do not have the same response to the treatment, i.e., some may respond well while others do not respond at all.

Cancer resistance to therapy can be primary, due to initially resistant disease, or secondary, occurring after a certain period in tumors that were primarily treatment-sensitive. Therefore, an assessment of tumor heterogeneity is crucial for the development of effective, targeted, and individualized therapies [60]. A personalized therapeutic approach may potentially target various tumor clones with a better therapeutic outcome compared to standard treatment options.

Regarding the DTC, its heterogeneity presents a clinical challenge, particularly taking into account the intratumor variability. DTC cell phenotypes are the product of a genotype, stochastic effects, and environmental factors. Genetic and epigenetic modulations occurring during cancer growth and progression result in different cell phenotypes [61]. The existence of intratumor heterogeneity is well known in advanced and metastatic thyroid DTC, and it can occur at any step of the cancer’s development, including early-stage tumors [62]. The high heterogeneity profile associated with these clinical stages may be responsible for the resistance to the theranostic approach and poor outcomes in some patients.

Intratumor heterogeneity presents an obstacle to revealing all the mechanisms of disease progression and adequate patient management. Furthermore, intratumor heterogeneity may be *spatial*, i.e., between different lesions, or *temporal*, which occurs between different lesions over time, representing additional clinical challenges [63].

All in all, as metastases in DTC may be present for a very long time, sometimes even decades, it may lead to the development of very different cell clones, which in turn result in a differential expression of theranostic targets (Figure 2 and Figure 3). Therefore, it is highly advisable to always include [^18^F]FDG imaging when considering theranostics in RAI-R DTC. [^18^F]FDG itself is not used as a theranostic tracer but may reveal lesions that may not respond to a personalized theranostic approach. It usually reveals highly aggressive DTC clones that may be treated with other local and/or systemic therapies. Novel cancer therapies need to be highly selective, targeting crucial genetic and epigenetic alterations. Due to the tumor escape mechanism usually occurring during long-term disease, therapies covering multiple cancer growth pathways are likely, at least in theory, to be more efficient compared to standard treatment options.

## 5. Available Evidence and Cost-Effectiveness

A significant body of evidence supports the use of [^18^F]FDG PET/CT for the localization of thyroid cancer lesions in patients with negative RAI imaging if residual disease is suspected. Indeed, solitary [^18^F]FDG-avid metastasis can be successfully treated by curative surgery. Moreover, the detection of disseminated [^18^F]FDG-avid metastasis may assist in performing a targeted biopsy if needed and suggesting systemic therapies. Finally, the determination of the tumor burden and the response assessment can support changes in treatment management. Cost-effectiveness data comparing [^18^F]FDG PET/CT to conventional imaging in detecting recurrent DTC are sparse [64,65]. Notably, the authors included all patients with a negative RAI scan (either diagnostic or post-therapy), and thyroglobulin levels were not considered. Vice versa, different clinical guidelines and the Society of Nuclear Medicine and Molecular Imaging Thyroid Carcinoma Appropriate Use Criteria [66] strongly support a patient-based use of [^18^F]FDG PET/CT, taking into account the histotype, the clinical history, the absolute thyroglobulin value, and the thyroglobulin doubling time [67]. This approach is able to restrict the number of patients submitted to the examination and optimize clinical accuracy [68]. Further cost-effectiveness studies on the selective use of [^18^F]FDG PET/CT in patients with suspected RAI-negative DTC recurrences are needed, but, for the moment, the procedure is relevant in clinical practice. Besides [^18^F]FDG, imaging with SSTR-ligands also showed good sensitivity in detecting RAI-negative and [^18^F]FDG-negative recurrences. Even if DTCs are not classified as neuroendocrine tumors, a relevant proportion of them show cellular expression of SSTRs independently from GLUT1 overexpression. Accordingly, some authors demonstrated in a small series of patients the good sensitivity of SSTR-ligand imaging in recurrent DTC, also allowing the detection of [^18^F]FDG-negative lesions [69,70]. Although there is sparse support in the literature, the Society of Nuclear Medicine and Molecular Imaging Thyroid Carcinoma Appropriate Use Criteria panel also supports the selective use of SSTR-ligand PET/CT in the presence of suspected RAI-negative recurrence and negative results of conventional imaging and [^18^F]FDG PET/CT [66]. Remarkably, no evidence is available to support other radiopharmaceuticals (PSMA analogs, FAP-targeting radiopharmaceuticals) illustrated in our present paper. They show promising results, but larger prospective studies are needed to confirm these data, and their use should be restricted, for now, to experimental trials. Another problem is the availability and cost of these tracers. SSTR and PSMA analogs are more widely available nowadays compared to novel FAP-targeting ones, which unfortunately have very limited availability across the world, and, finally, we lack complete cost-effectiveness data for these tracers.

## 6. Conclusions

In RAI-R DTC, the role of [^18^F]FDG PET/CT imaging is well-established for diagnostic and prognostic purposes. The role of SSTR should be considered by the treating team in highly selected cases when other imaging procedures are not conclusive. New promising radiopharmaceuticals, PSMA, and FAP-targeting tracers were recently tested in DTC patients and opened new diagnostic and therapeutic perspectives in the field. Their role, however, is not completely clarified, and well-designed studies are warranted to evaluate their potential in critical RAI-R DTC patients.

Still, these tracers offer the potential for a theranostic approach, which is of incremental value in RAI-R DTC patients who underwent progression despite standard treatment regimens.

## Figures and Tables

**Table 1:** Criteria to define radioiodine-refractory differentiated thyroid cancer.

**Main Criteria**
No ^131^I uptake in malignant metastatic tissue outside the thyroid bed on the first post-therapeutic WBS
^131^I uptake is lost after previous evidence of ^131^I avid disease
^131^I is concentrated in some but not in other lesions
Disease progresses despite ^131^I avidity
Patients have already received 22 GBq or more of ^131^I
**Other Potential Criteria**
No ^131^I uptake in malignant tissue on diagnostic WBS
Significant uptake on [^18^F]FDG PET/CT
Aggressive tumor histology

Legend: WBS—whole body scan; GBq—Gigabecquerel; [^18^F]FDG—2-[^18^F]fluoro-2-deoxy-D-glucose; PET/CT—positron emission tomography/computed tomography.

## Data Availability

Data sharing not applicable.

## References

[1] Giovanella L., Deandreis D., Vrachimis A., Campenni A., Ovcaricek P.P. (2022). Molecular Imaging and Theragnostics of Thyroid Cancers. Cancers.

[2] Schlumberger M., Brose M., Elisei R., Leboulleux S., Luster M., Pitoia F., Pacini F. (2014). Definition and Management of Radioactive Iodine-Refractory Differentiated Thyroid Cancer. Lancet Diabetes Endocrinol..

[3] Haugen B.R., Alexander E.K., Bible K.C., Doherty G.M., Mandel S.J., Nikiforov Y.E., Pacini F., Randolph G.W., Sawka A.M., Schlumberger M. (2016). 2015 American Thyroid Association Management Guidelines for Adult Patients with Thyroid Nodules and Differentiated Thyroid Cancer: The American Thyroid Association Guidelines Task Force on Thyroid Nodules and Differentiated Thyroid Cancer. Thyroid.

[4] Agrawal N., Akbani R., Aksoy B.A., Ally A., Arachchi H., Asa S.L., Auman J.T., Balasundaram M., Balu S., Baylin S.B. (2014). Integrated Genomic Characterization of Papillary Thyroid Carcinoma. Cell.

[5] Lim H., Devesa S.S., Sosa J.A., Check D., Kitahara C.M. (2017). Trends in Thyroid Cancer Incidence and Mortality in the United States, 1974-2013. JAMA.

[6] Vaisman F., Carvalho D.P., Vaisman M. (2015). A New Appraisal of Iodine Refractory Thyroid Cancer. Endocr. Relat. Cancer.

[7] Van Nostrand D. (2018). Radioiodine Refractory Differentiated Thyroid Cancer: Time to Update the Classifications. Thyroid.

[8] Ylli D., Van Nostrand D., Wartofsky L. (2019). Conventional Radioiodine Therapy for Differentiated Thyroid Cancer. Endocrinol. Metab. Clin. N. Am..

[9] Kim H., Kim H.I., Kim S.W., Jung J., Jeon M.J., Kim W.G., Kim T.Y., Kim H.K., Kang H.C., Han J.M. (2018). Prognosis of Differentiated Thyroid Carcinoma with Initial Distant Metastasis: A Multicenter Study in Korea. Endocrinol. Metab..

[10] Van Nostrand D. (2016). 131I Treatment of Distant Metastases. Thyroid Cancer: A Comprehensive Guide to Clinical Management.

[11] Giovanella L., Van Nostrand D. (2019). Advanced Differentiated Thyroid Cancer: When to Stop Radioiodine?. Q. J. Nucl. Med. Mol. Imaging.

[12] Sacks W., Braunstein G.D. (2014). Evolving Approaches in Managing Radioactive Iodine-Refractory Differentiated Thyroid Cancer. Endocr. Pract..

[13] Luo Y., Jiang H., Xu W., Wang X., Ma B., Liao T., Wang Y. (2020). Clinical, Pathological, and Molecular Characteristics Correlating to the Occurrence of Radioiodine Refractory Differentiated Thyroid Carcinoma: A Systematic Review and Meta-Analysis. Front. Oncol..

[14] Hänscheid H., Lassmann M., Buck A.K., Reiners C., Verburg F.A. (2014). The Limit of Detection in Scintigraphic Imaging with I-131 in Patients with Differentiated Thyroid Carcinoma. Phys. Med. Biol..

[15] Lee J.W., Lee S.M., Koh G.P., Lee D.H. (2011). The Comparison of (131)I Whole-Body Scans on the Third and Tenth Day after (131)I Therapy in Patients with Well-Differentiated Thyroid Cancer: Preliminary Report. Ann. Nucl. Med..

[16] Avram A.M., Giovanella L., Greenspan B., Lawson S.A., Luster M., Van Nostrand D., Peacock J.G., Ovčariček P.P., Silberstein E., Tulchinsky M. (2022). SNMMI Procedure Standard/EANM Practice Guideline for Nuclear Medicine Evaluation and Therapy of Differentiated Thyroid Cancer: Abbreviated Version. J. Nucl. Med..

[17] Sakulpisuti C., Charoenphun P., Chamroonrat W. (2022). Positron Emission Tomography Radiopharmaceuticals in Differentiated Thyroid Cancer. Molecules.

[18] Heydarzadeh S., Moshtaghie A.A., Daneshpoor M., Hedayati M. (2020). Regulators of Glucose Uptake in Thyroid Cancer Cell Lines. Cell Commun. Signal..

[19] Feine U., Lietzenmayer R., Hanke J.P., Wohrle H., Muller-Schauenburg W. (1995). 18FDG Whole-Body PET in Differentiated Thyroid Carcinoma. Flipflop in Uptake Patterns of 18FDG and 131I. Nuklearmedizin.

[20] Feine U., Lietzenmayer R., Hanke J.P., Held J., Wöhrle H., Müller-Schauenburg W. (1996). Fluorine-18-FDG and Iodine-131-Iodide Uptake in Thyroid Cancer. J. Nucl. Med..

[21] Liu M., Cheng L., Jin Y., Ruan M., Sheng S., Chen L. (2018). Predicting 131I-Avidity of Metastases from Differentiated Thyroid Cancer Using 18F-FDG PET/CT in Postoperative Patients with Elevated Thyroglobulin. Sci. Rep..

[22] Treglia G., Goichot B., Giovanella L., Hindié E., Jha A., Pacak K., Taïeb D., Walter T., Imperiale A. (2020). Prognostic and Predictive Value of Nuclear Imaging in Endocrine Oncology. Endocrine.

[23] Deandreis D., Al Ghuzlan A., Leboulleux S., Lacroix L., Garsi J.P., Talbot M., Lumbroso J., Baudin E., Caillou B., Bidart J.M. (2011). Do Histological, Immunohistochemical, and Metabolic (Radioiodine and Fluorodeoxyglucose Uptakes) Patterns of Metastatic Thyroid Cancer Correlate with Patient Outcome?. Endocr. Relat. Cancer.

[24] Manohar P.M., Beesley L.J., Bellile E.L., Worden F.P., Avram A.M. (2018). Prognostic Value of FDG-PET/CT Metabolic Parameters in Metastatic Radioiodine-Refractory Differentiated Thyroid Cancer. Clin. Nucl. Med..

[25] Albano D., Dondi F., Mazzoletti A., Bellini P., Rodella C., Bertagna F. (2021). Prognostic Role of 2-[18F]FDG PET/CT Metabolic Volume Parameters in Patients Affected by Differentiated Thyroid Carcinoma with High Thyroglobulin Level, Negative 131I WBS and Positive 2-[18F]-FDG PET/CT. Diagnostics.

[26] Ha L.N., Iravani A., Nhung N.T., Hanh N.T.M., Hutomo F., Son M.H. (2021). Relationship between Clinicopathologic Factors and FDG Avidity in Radioiodine-Negative Recurrent or Metastatic Differentiated Thyroid Carcinoma. Cancer Imaging.

[27] Oh J.M., Ahn B.C. (2021). Molecular Mechanisms of Radioactive Iodine Refractoriness in Differentiated Thyroid Cancer: Impaired Sodium Iodide Symporter (NIS) Expression Owing to Altered Signaling Pathway Activity and Intracellular Localization of NIS. Theranostics.

[28] Santhanam P., Khthir R., Solnes L.B., Ladenson P.W. (2018). THE RELATIONSHIP OF BRAFV600E MUTATION STATUS TO FDG PET/CT AVIDITY IN THYROID CANCER: A REVIEW AND META-ANALYSIS. Endocr. Pract..

[29] Chan H.P., Chen I.F., Tsai F.R., Kao C.H., Shen D.H.Y. (2023). Reversing “Flip-Flop” Phenomenon of 131 I and Glucose Avidity in RET-Fusion Positive Radioiodine-Refractory Thyroid Cancer Lesions After Treatment of Pralsetinib. Clin. Nucl. Med..

[30] Weber M., Kersting D., Riemann B., Brandenburg T., Führer-Sakel D., Grünwald F., Kreissl M.C., Dralle H., Weber F., Schmid K.W. (2022). Enhancing Radioiodine Incorporation into Radioiodine-Refractory Thyroid Cancer with MAPK Inhibition (ERRITI): A Single-Center Prospective Two-Arm Study. Clin. Cancer Res..

[31] Leboulleux S., Schroeder P.R., Busaidy N.L., Auperin A., Corone C., Jacene H.A., Ewertz M.E., Bournaud C., Wahl R.L., Sherman S.I. (2009). Assessment of the Incremental Value of Recombinant Thyrotropin Stimulation before 2-[18F]-Fluoro-2-Deoxy-D-Glucose Positron Emission Tomography/Computed Tomography Imaging to Localize Residual Differentiated Thyroid Cancer. J. Clin. Endocrinol. Metab..

[32] Israeli R.S., Powell C.T., Fair W.R., Heston W.D.W. (1993). Molecular Cloning of a Complementary DNA Encoding a Prostate-Specific Membrane Antigen. Cancer Res..

[33] Ciappuccini R., Saguet-Rysanek V., Giffard F., Licaj I., Dorbeau M., Clarisse B., Poulain L., Bardet S. (2021). PSMA Expression in Differentiated Thyroid Cancer: Association With Radioiodine, 18FDG Uptake, and Patient Outcome. J. Clin. Endocrinol. Metab..

[34] Heitkötter B., Steinestel K., Trautmann M., Grünewald I., Barth P., Gevensleben H., Bögemann M., Wardelmann E., Hartmann W., Rahbar K. (2018). Neovascular PSMA Expression Is a Common Feature in Malignant Neoplasms of the Thyroid. Oncotarget.

[35] Sollini M., di Tommaso L., Kirienko M., Piombo C., Erreni M., Lania A.G., Erba P.A., Antunovic L., Chiti A. (2019). PSMA Expression Level Predicts Differentiated Thyroid Cancer Aggressiveness and Patient Outcome. EJNMMI Res..

[36] Sanchez-Crespo A. (2013). Comparison of Gallium-68 and Fluorine-18 Imaging Characteristics in Positron Emission Tomography. Appl. Radiat. Isot..

[37] Martiniova L., De Palatis L., Etchebehere E., Ravizzini G. (2016). Gallium-68 in Medical Imaging. Curr. Radiopharm..

[38] Piron S., Verhoeven J., Vanhove C., De Vos F. (2022). Recent Advancements in 18F-Labeled PSMA Targeting PET Radiopharmaceuticals. Nucl. Med. Biol..

[39] Seifert R., Telli T., Opitz M., Barbato F., Berliner C., Nader M., Umutlu L., Stuschke M., Hadaschik B., Herrmann K. (2023). Unspecific 18F-PSMA-1007 Bone Uptake Evaluated Through PSMA-11 PET, Bone Scanning, and MRI Triple Validation in Patients with Biochemical Recurrence of Prostate Cancer. J. Nucl. Med..

[40] Grünig H., Maurer A., Thali Y., Kovacs Z., Strobel K., Burger I.A., Müller J. (2021). Focal Unspecific Bone Uptake on [18F]-PSMA-1007 PET: A Multicenter Retrospective Evaluation of the Distribution, Frequency, and Quantitative Parameters of a Potential Pitfall in Prostate Cancer Imaging. Eur. J. Nucl. Med. Mol. Imaging.

[41] Arnfield E.G., Thomas P.A., Roberts M.J., Pelecanos A.M., Ramsay S.C., Lin C.Y., Latter M.J., Garcia P.L., Pattison D.A. (2021). Clinical Insignificance of [18F]PSMA-1007 Avid Non-Specific Bone Lesions: A Retrospective Evaluation. Eur. J. Nucl. Med. Mol. Imaging.

[42] Duncan I., Ingold N., Martinez-Marroquin E., Paterson C. (2023). An Australian Experience Using Tc-PSMA SPECT/CT in the Primary Diagnosis of Prostate Cancer and for Staging at Biochemical Recurrence after Local Therapy. Prostate.

[43] Rizzo A., Racca M., Dall’Armellina S., Delgado Bolton R.C., Albano D., Dondi F., Bertagna F., Annunziata S., Treglia G. (2023). Potential Role of PSMA-Targeted PET in Thyroid Malignant Disease: A Systematic Review. Diagnostics.

[44] De Vries L.H., Lodewijk L., Braat A.J.A.T., Krijger G.C., Valk G.D., Lam M.G.E.H., Borel Rinkes I.H.M., Vriens M.R., de Keizer B. (2020). 68Ga-PSMA PET/CT in Radioactive Iodine-Refractory Differentiated Thyroid Cancer and First Treatment Results with 177Lu-PSMA-617. EJNMMI Res..

[45] Wächter S., Di Fazio P., Maurer E., Manoharan J., Keber C., Pfestroff A., Librizzi D., Bartsch D.K., Luster M., Eilsberger F. (2021). Prostate-Specific Membrane Antigen in Anaplastic and Poorly Differentiated Thyroid Cancer-A New Diagnostic and Therapeutic Target?. Cancers.

[46] Sartor O., de Bono J., Chi K.N., Fizazi K., Herrmann K., Rahbar K., Tagawa S.T., Nordquist L.T., Vaishampayan N., El-Haddad G. (2021). Lutetium-177–PSMA-617 for Metastatic Castration-Resistant Prostate Cancer. N. Engl. J. Med..

[47] Johnbeck C.B., Knigge U., Kjær A. (2014). PET Tracers for Somatostatin Receptor Imaging of Neuroendocrine Tumors: Current Status and Review of the Literature. Future Oncol..

[48] Pazaitou-Panayiotou K., Janson E.T., Koletsa T., Kotoula V., Stridsberg M., Karkavelas G., Karayannopoulou G. (2012). Somatostatin Receptor Expression in Non-Medullary Thyroid Carcinomas. Hormones.

[49] Ain K.B., Taylor K.D., Tofiq S., Venkataraman G. (1997). Somatostatin Receptor Subtype Expression in Human Thyroid and Thyroid Carcinoma Cell Lines. J. Clin. Endocrinol. Metab..

[50] Ocak M., Demirci E., Kabasakal L., Aygun A., Tutar R.O., Araman A., Kanmaz B. (2013). Evaluation and Comparison of Ga-68 DOTA-TATE and Ga-68 DOTA-NOC PET/CT Imaging in Well-Differentiated Thyroid Cancer. Nucl. Med. Commun..

[51] Versari A., Sollini M., Frasoldati A., Fraternali A., Filice A., Froio A., Asti M., Fioroni F., Cremonini N., Putzer D. (2014). Differentiated Thyroid Cancer: A New Perspective with Radiolabeled Somatostatin Analogues for Imaging and Treatment of Patients. Thyroid..

[52] Maghsoomi Z., Emami Z., Malboosbaf R., Malek M., Khamseh M.E. (2021). Efficacy and Safety of Peptide Receptor Radionuclide Therapy in Advanced Radioiodine-Refractory Differentiated Thyroid Cancer and Metastatic Medullary Thyroid Cancer: A Systematic Review. BMC Cancer.

[53] Lee D.Y., Kim Y.I. (2020). Peptide Receptor Radionuclide Therapy in Patients With Differentiated Thyroid Cancer: A Meta-Analysis. Clin. Nucl. Med..

[54] Budiawan H., Salavati A., Kulkarni H.R., Baum R.P. (2014). Peptide Receptor Radionuclide Therapy of Treatment-Refractory Metastatic Thyroid Cancer Using 90Yttrium and 177Lutetium Labeled Somatostatin Analogs: Toxicity, Response and Survival Analysis. Am. J. Nucl. Med. Mol. Imaging.

[55] Giesel F.L., Kratochwil C., Lindner T., Marschalek M.M., Loktev A., Lehnert W., Debus J., Jäger D., Flechsig P., Altmann A. (2019). 68Ga-FAPI PET/CT: Biodistribution and Preliminary Dosimetry Estimate of 2 DOTA-Containing FAP-Targeting Agents in Patients with Various Cancers. J. Nucl. Med..

[56] Chen Y., Zheng S., Zhang J., Yao S., Miao W. (2022). 68Ga-DOTA-FAPI-04 PET/CT Imaging in Radioiodine-Refractory Differentiated Thyroid Cancer (RR-DTC) Patients. Ann. Nucl. Med..

[57] Fu H., Fu J., Huang J., Pang Y., Chen H. (2021). 68Ga-FAPI PET/CT Versus 18F-FDG PET/CT for Detecting Metastatic Lesions in a Case of Radioiodine-Refractory Differentiated Thyroid Cancer. Clin. Nucl. Med..

[58] Ballal S., Yadav M.P., Moon E.S., Roesch F., Kumari S., Agarwal S., Tripathi M., Sahoo R.K., Mangu B.S., Tupalli A. (2022). Novel Fibroblast Activation Protein Inhibitor-Based Targeted Theranostics for Radioiodine-Refractory Differentiated Thyroid Cancer Patients: A Pilot Study. Thyroid.

[59] Affinito O., Orlandella F.M., Luciano N., Salvatore M., Salvatore G., Franzese M. (2022). Evolution of Intra-Tumoral Heterogeneity across Different Pathological Stages in Papillary Thyroid Carcinoma. Cancer Cell Int..

[60] Dagogo-Jack I., Shaw A.T. (2017). Tumour Heterogeneity and Resistance to Cancer Therapies. Nat. Rev. Clin. Oncol..

[61] Hu J., Yuan I.J., Mirshahidi S., Simental A., Lee S.C., Yuan X. (2021). Thyroid Carcinoma: Phenotypic Features, Underlying Biology and Potential Relevance for Targeting Therapy. Int. J. Mol. Sci..

[62] Ieni A., Vita R., Pizzimenti C., Benvenga S., Tuccari G. (2021). Intratumoral Heterogeneity in Differentiated Thyroid Tumors: An Intriguing Reappraisal in the Era of Personalized Medicine. J. Pers. Med..

[63] Bedard P.L., Hansen A.R., Ratain M.J., Siu L.L. (2013). Tumour Heterogeneity in the Clinic. Nature.

[64] Biz A., Schluckebier L., Bastos C., Silva R., Braga J., Caetano R. (2015). Cost-Effectiveness of The Use of 18fdg-Pet/Ct In The Detection of Recurrent Differentiated Thyroid Cancer. Value Health.

[65] Khiewvan B., Nopmaneejumruslers C., Pusuwan P., Tuchinda P., Tojinda N., Ubolnuch K. (2013). Cost-Effectiveness Analysis of 18F-FDG PET/CT in Detecting Suspected Recurrence or Metastasis in Well-Differentiated Thyroid Carcinoma Patients with Negative Diagnostic Total Body Scan in Thailand: A Decision Analysis. J. Med. Assoc. Thai.

[66] Donohoe K.J., Aloff J., Avram A.M., Bennet K.G., Giovanella L., Greenspan B., Gulec S., Hassan A., Kloos R.T., Solorzano C.C. (2020). Appropriate Use Criteria for Nuclear Medicine in the Evaluation and Treatment of Differentiated Thyroid Cancer. J. Nucl. Med..

[67] Giovanella L., Trimboli P., Verburg F.A., Treglia G., Piccardo A., Foppiani L., Ceriani L. (2013). Thyroglobulin Levels and Thyroglobulin Doubling Time Independently Predict a Positive 18F-FDG PET/CT Scan in Patients with Biochemical Recurrence of Differentiated Thyroid Carcinoma. Eur. J. Nucl. Med. Mol. Imaging.

[68] Araz M., Soydal Ç., Özkan E., Akkuş P., Nak D., Özlem Küçük N., Metin Klr K. (2021). Role of Thyroglobulin Doubling Time in Differentiated Thyroid Cancer and Its Relationship with Demographic-Histopathologic Risk Factors and 18F-Fluorodeoxyglucose Positron Emission Tomography/Computed Tomography Parameters. Cancer Biother. Radiopharm..

[69] Middendorp M., Selkinski I., Happel C., Kranert W.T., Grünwald F. (2010). Comparison of Positron Emission Tomography with [(18)F]FDG and [(68)Ga]DOTATOC in Recurrent Differentiated Thyroid Cancer: Preliminary Data. Q. J. Nucl. Med. Mol. Imaging.

[70] Vrachimis A., Stegger L., Wenning C., Noto B., Burg M.C., Konnert J.R., Allkemper T., Heindel W., Riemann B., Schäfers M. (2016). [68Ga]DOTATATE PET/MRI and [18F]FDG PET/CT Are Complementary and Superior to Diffusion-Weighted MR Imaging for Radioactive-Iodine-Refractory Differentiated Thyroid Cancer. Eur. J. Nucl. Med. Mol. Imaging.

